# Suitability of methods for *Plasmodium falciparum* cultivation in atmospheric air

**DOI:** 10.1590/0074-02760210331

**Published:** 2022-07-18

**Authors:** Marcell Crispim, Ignasi Bofill Verdaguer, Sofia Ferreira Silva, Alejandro Miguel Katzin

**Affiliations:** 1 Universidade de São Paulo, Instituto de Ciências Biomédicas, Departamento de Parasitologia, São Paulo, SP, Brasil

**Keywords:** Plasmodium falciparum, culture, atmospheric air, drugs, parasite viability

## Abstract

**BACKGROUND:**

One of the most controversial factors about malaria parasite culture is the gaseous composition used. The most commonly used one consists of a mixture poor in O_2_ and rich in CO_2_.

**OBJECTIVES:**

The present study aimed to share standard methods from our research group simplifying *Plasmodium falciparum* cultures by employing atmospheric air (ATM) and reusable glass bottles under agitation.

**METHODS:**

Here, it was compared the parasite viability, free oxygen in media, and drug sensitivity between different strains and isolates maintained for long periods under ATM or classic conditions.

**FINDINGS:**

The oxygen concentration in media under ATM was slightly superior to that observed in human blood and the media under the classic gaseous mixture. However, ATM or the use of glass bottles did not affect parasitic proliferation after several years of culture. Noticeably, the introduction of ATM altered reversibly the efficacy of several antimalarials. This influence was different between the strains and isolate.

**CONCLUSIONS:**

ATM conditions and shaken flasks could be used as a standard method condition for culture manutention since they do not differ greatly from classical 5% O_2_ gas mixtures in terms of parasite proliferation and do not impose non-reversible changes to *P. falciparum* physiology.

Malaria is a prevalent parasitic disease in Africa, Southeast Asia, and South America, affecting 241 million people in 2019 and has caused approximately 627,000 deaths mostly due to *Plasmodium falciparum* infections in Africa.^1^ Moreover, malaria control presents other challenges including the major issue of resistance drug resistance.^1^ ^,^ ^2^ That is why the discovery of new antimalarial compounds, knowledge of the parasite’s metabolism, and assessment of the drug-resistance spreading are required.^1^ ^,^ ^2^ Consequently, interdisciplinary efforts are needed to fight against malaria on a global scale.

Central to the study of infectious diseases is the *in vitro* culturing of the etiologic agent. The creation of an artificial, continuous intraerythrocytic cycle *ex vivo* was described in 1976 by two groups simultaneously, Trager and Jensen^3^ and Haynes et al.^4^ Nowadays, the Trager and Jensen method is largely the most employed in research. Epidemiologic studies focused on adapting isolates to culture employ this method^5^ for *in vitro* drug screening, the study of the parasitic metabolism, and for studies requiring large amounts of parasites.

The Trager and Jensen culture methodology was based on the use of candle jars or cell flasks containing human red blood cells suspended in a culture medium, human serum, and a specific gaseous mixture rich in CO_2_ and poor in O_2_.^3^ These gaseous conditions were obtained by direct gas injection or by candle combustion.^3^ Some authors optimised the initial culture methodology: the most important modifications were the substitution of human plasma for commercial Lipid-Rich Bovine serum albumin^6^ and the parasitic stage synchronisation by the D-sorbitol techniques.^7^ Preechapornkul et al. in 2010, used bioreactors under constant agitation and a controlled atmosphere to optimise the *P. falciparum* culture.^8^ Moreover, Radfar et al. combined new techniques and standardised very intricate protocols to scale up the culturing for large-scale parasite recovery.^9^ Finally, Moloney et al. in 1990 developed culture conditions to enable the asexual erythrocytic stage of *P. falciparum* to be grown in deep culture in reusable bottles. Thus, the authors published a protocol that made it easier and less expensive to cultivate large volumes of parasites in a single vessel (up to eight litres).^10^

An important requirement for *in vitro* culture is the ability to mimic physiologic conditions such as temperature changes, immune responses, and gaseous composition.^11^ Different gaseous mixtures and atmospheric air (ATM) were employed across the studies and marginal effects on the parasitic viability were observed.^12^ ^,^ ^13^ The only related effect of modifying gaseous conditions is an increase in the duration of schizogony in parasites exposed to 21% O_2_.^13^ However, parasites cultured at high oxygen tensions showed to possess different susceptibility to some antimalarial drugs.^13^ ^,^ ^14^ For example, Briolant et al., showed that chloroquine (CQ) IC_50_ value at 10% O_2_ was significantly higher than several isolates maintained at 21% O_2_.^13^ Importantly, ~ 30% of the isolates that were *in vitro* resistant to CQ at 10% O_2_ by considering an IC_50_ > 100 nM, become sensitive at 21% O2 (IC_50_ < 100 nM). The results make the authors suggest a standardised *in vitro* assay protocol to survey malaria drug resistance.

Here we present some standard methods from our research group that may provide easier and cheaper *Plasmodium* cultures suitable for small- or large-scale use. These methods simplify the classic methodology by employing ATM and by maintaining parasites in reusable glass bottles under agitation. These techniques are a combination of others already described but their use together had not been reported yet. For that reason, we did experiments to assess the impact of the gas and physical conditions in culture; specifically, we focused on growth rates and drug susceptibility. Moreover, we discuss different culturing methods in the literature regarding their applicability to laboratory use and to whether they mimic reasonably the natural environment of the parasite.

## MATERIALS AND METHODS

*Reagents* - Albumax I and RPMI-1640 were purchased from Thermo Fisher Scientific (Waltham, MA). A gaseous mixture of 5% CO_2_, 5% O_2_, and 90% N_2_ was purchased from Air Products Brasil LTDA (São Paulo, SP, Brazil). Other reagents include saponin, hypoxanthine, gentamycin sulfate, D-sorbitol, glucose, 4-(2-hydroxyethyl)-1-piperazine ethane sulfonic acid (HEPES), atovaquone (AV), and chloroquine (CQ) were purchased from Sigma-Aldrich (St. Louis, MO). Artesunate (ART) and SYBR Green I^®^ were purchased from the Guiling Pharmaceutical Factory (Guiling City, 21 Guangxi, China) and Thermo Fisher Scientific (Waltham, MA) respectively.

**Plasmodium falciparum intraerythrocytic stages culture**

*Basic requirements for Plasmodium culture* - Throughout this work, we used one malaria parasite isolate: NF54 (non-cloned drug sensible isolate obtained from one case of malaria imported into the Netherlands)^15^ and two different *P. falciparum* culture adapted strain clones: K1 (isolated in India and reported to be CQ-resistant)^16^ and 3D7 (a clone derived from NF54 by limiting dilution).^17^ Parasites were cultured at 37ºC in RPMI-1640 medium dissolved in ultra-pure in Milli-Q water (Millipore, Burlington, MA) and supplemented with 25 mM HEPES, 0.5% Albumax I^®^, 370 μM hypoxanthine, 2 g/L glucose, and 25 mg/L gentamicin sulfate.^9^ The pH of the media was adjusted to 7.4 with a 10% NaHCO_3_ sterile solution and then it was filtered in Millipore Express^TM^ PLUS 0.22 µm sterile filters.

The reference gaseous mixture was composed of 5% CO_2_, 5% O_2_, and 90% N_2_,^9^ however, ATM was also employed when indicated. To avoid culture contamination, mycoplasma testing was done regularly and optic microscopy was performed daily.^18^ Leukodepleted blood (blood type A, Rh-positive) was a gift from Sírio Libanês Hospital (São Paulo, SP, Brazil) blood bank.

*Trager & Jensen-based culture* - The *P. falciparum* intraerythrocytic stages at 2% haematocrit were cultured *in vitro* at 37ºC according to Trager & Jensen^3^ with modifications^9^ in RPMI-1640 complete culture medium in 25 or 75 cm^3^ sterile cell culture flasks, containing 5 and 20 mL of culture respectively, purchased from Corning^®^ (Corning, NY, EUA), filled up as described in next item. Culture changes were performed as described by Radfar et al.^9^ and the reference gaseous mixture or ATM was employed when indicated. The cap was screwed tightly after infecting the gas mixture or allowing ATM into the flasks. The culture medium was changed once every two days if parasitaemia < 5% and every day if parasitaemia ≥ 5%.

*Culture in glass bottles* - Parasites were cultured at 37ºC in 2, 1, 0.5, 0.25, or 0.1 L hermetic and sterile glass bottles (Schott Glas, Mainz, Germany). 20% of the bottle’s total volume was filled up with *Plasmodium* culture. The culture was adjusted to a 2% haematocrit culture as described. Culture changes were performed as described by Radfar et al.^9^ and the reference gaseous mixture or ATM was employed when indicated. The cultures were maintained at 37ºC and under orbital agitation (130 RPM) in a MaxQ 6000^®^ shaker model 4353 from Thermo Fisher Scientific (Waltham, MA). In this methodology, ATM was employed by filling the space of the bottle. The medium was removed from the culture by centrifugation.

*Parasitic synchronisation at the ring stage* - Culture synchronisation at the ring stage (6-22 h after the invasion) was performed as described by C. Lambros & Vanderberg.^7^ Briefly, cultures were centrifuged at 200 x *g* for 5 min and synchronisation was performed by incubating the pellet in a 5% D-sorbitol (w/v) approximately 0.5 mL red blood cells/2.5 mL D-sorbitol solution).

*Drug assays* - Drug experiments that required the measurement of parasite proliferation were performed in 96-well plates contained in hermetic boxes for plate incubation. The boxes were filled with the indicated gaseous mixture or maintained in ATM. All assays started at the ring stage (2% parasitaemia, 2% haematocrit) and parasite growth was monitored after 48 h by both Giemsa-stained smears and DNA staining by SYBR^TM^ Green I.^19^ The compound concentration which decreases at 50% of parasitic growth (IC_50_) at 48 h was calculated following the Smilkstein et al. methodology.^19^ Briefly, 1:1 (vol: vol) serial dilution was performed to obtain 19 decreasing drug concentrations in the 96-well plate. An initial concentration of 400 nM for AV and 1000 nM for CQ or ART was selected. Comparison with untreated controls and a solvent control was always performed.

*Parasitic growth monitoring* - Growth was monitored after 48 h by Giemsa stained smears by optical microscopy or DNA staining for all experiments. For DNA staining, we followed procedures described by Smilkstein et al.^19^ An amount of 100 μL of parasite culture was incubated in a 96-well cell plate in darkness and at room temperature after the addition of 100 μL of SYBR^TM^ Green I 2/10.000 (v/v) in lysis buffer [20 mM Tris, pH 7.5; 5 mM EDTA; 0.008% saponin (w/v); 0.08% Triton X-100 (v/v)]. We used uninfected erythrocytes as blanks at the same haematocrit and subtracted its fluorescence measurements from the readings of the samples. Fluorescence was measured using POLARstar Omega fluorometer^®^ (BMG Labtech^®^, Ortenberg, Germany) at the excitation and emission wavelengths of 485 and 520 nm, respectively.

*Oxygraphy* - Red blood cells were maintained at 2% haematocrit in 25 cm^3^ sterile cell culture flasks, containing 5 mL of complete RPMI medium. The flasks were incubated with a classic gaseous mixture or ATM for 24 h at 37ºC. Then, the sample was homogenised and the oxygen content in 2 mL was assessed at 37ºC in a high-resolution oxygraph (Oxygraph-2k Oroborus Instruments, Innsbruck, Austria).

*Statistical analyses* - Parasitic proliferation was studied by microscopy of Giemsa-stained smears and statistically analysed to compare the parasitic growth/stages under different culture methodologies or conditions by a one-way ANOVA/Tukey multiple comparison test. Parasitic proliferation was studied by microscopy of Giemsa-stained smears and statistically analysed to compare the parasitic growth/stages under different culture methodologies or conditions by a one-way ANOVA/Tukey multiple comparison test.

The IC_50_ value at 48 h was analysed concerning the logarithm of the concentration of the compound using nonlinear regression (dose-response slope/variable sigmoid equation) using GraphPad Prism^®^ software (GraphPad Software, San Diego, CA). The R-squared value (R^2^) was also calculated for the dose-response and only those assays showing R^2^ > 0.90 were accepted. Statistical analyses between the IC_50_ values here presented were performed using a nonparametric Unpaired T-test. All experiments were independently repeated three times, using plates from different batches. Each experiment contained three parallel technical replicates.

## RESULTS

**Fig. 1:**
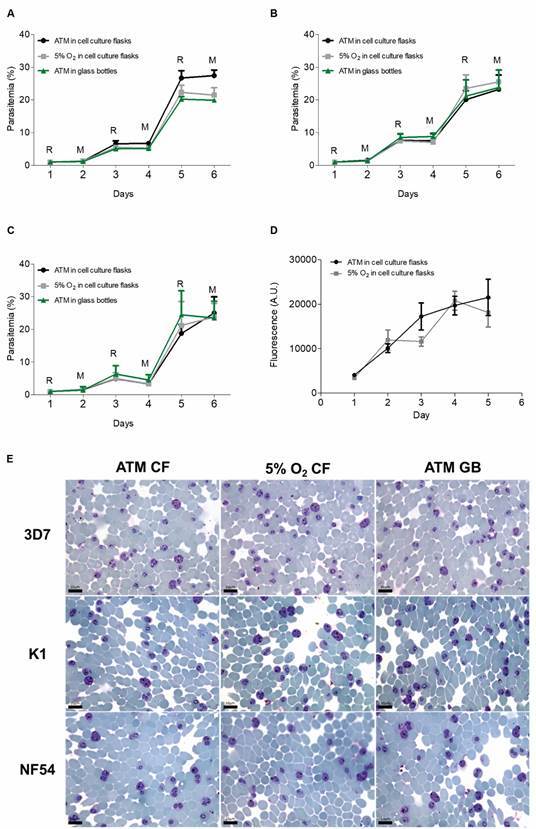
parasite proliferation under different culture conditions. Cycle progression of parasites under different gas conditions employing cell culture flasks or 0.1 L sterile glass bottles. The *Plasmodium falciparum* isolates (A) 3D7, (B) K1, and (C) NF54 were pre-cultivated and synchronised. 2% of haematocrit were infected reaching 1% parasitaemia (day 1). Parasitaemia was measured daily using optical microscopy as previously described. In (D) the culture parasitaemia of the 3D7 isolate was measured daily using the methodology previously described by Smilkstein et al.^19^ ATM: atmospheric air, final O_2_ concentration: 180.54 ± 4.14 µM. 5% O_2_: low-oxygen gaseous mixture described by Trager and Jensen, final O_2_ concentration: 147.36 ± 0.25 µM. R: ring/young trophozoite forms; M: mature trophozoite/schizont forms. In (E) are presented representative pictures of *P. falciparum* cultures of 3D7, K1, and NF54 strains at day 6 post-infection using optical microscopy. ATM CF: parasites cultivated using culture flasks under atmospheric air; 5% O_2_ CF: parasites were cultivated using culture flasks under a classical mixture of gas described by Trager and Jensen. ATM GB: parasites cultivated using glass bottles under atmospheric air. Cultures at 2% hematocrit were infected with 1% parasitaemia (day 1). All experiments were independently repeated three times.

*Influence of gas mixtures and cultivation method on parasite growth in vitro* - The strain 3D7 was cultured for one year in ATM before starting the experiments and the K1 strain and the NF54 isolate for three months. By employing these cultures, the following assays were performed. First, parasites were cultured in cell flasks or glass bottles under the classical gas mixture^3^ or ATM. Then, the parasitaemia of the cultures was monitored for at least 5 days (Fig. 1A-D). While starting at a parasitaemia of 1% we observed no differences in *P. falciparum* proliferation rate or the proportion of intraerythrocytic stages between cultures done in culture flasks with ATM or with 5% O_2_. It wasn’t also observed differences in parasite proliferation or the proportion of intraerythrocytic stages. Except for panel D, all data in Fig. 1 were obtained employing 0.1 L bottles, but similar results were obtained using 2, 1, 0.5, 0.25 L bottles (data not shown). Noticeably, cultures maintained without agitation in glass bottles did not allow parasite proliferation, and smaller ratios of parasitic growth were only observed if employing initial parasitaemia < 0.5% (data not shown).

**Fig. 2:**
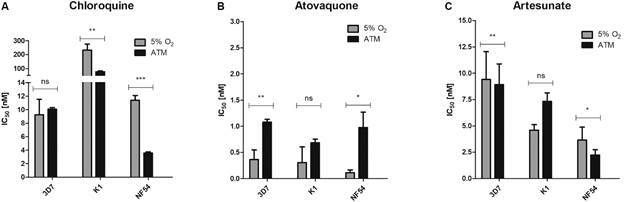
effect of gaseous mixtures on IC_50_ values for antimalarial agents. Effect of (A) chloroquine (CQ), (B) atovaquone (AV) and (C) artesunate (ART) in 3D7 and K1 strains and NF54 isolate. IC_50_ values were calculated as described and the experiments were repeated three times for each strain and antimalarial agent. The IC_50_ values of each drug under 5% O2 and atmospheric air (ATM) were compared and analysed using the Unpaired t-test. ***p < 0.001; **p < 0.01; *p < 0.05.

*Influence of O*_*2*_ *tension on antimalarial drug activity in vitro* - Free oxygen levels in erythrocytes under ATM (~180 µM) were slightly greater than those under 5% O_2_ (~147 µM) and consequently we verified if this difference could influence antimalarial drug activity *in vitro*. For this, parasites maintained in culture flasks under ATM conditions were used for *in vitro* drug screening assays. 96-well plates were filled with 200 µL of culture and were prepared as described and introduced in hermetic boxes containing ATM or 5% O_2_. We observed that under ATM there is no statistically significant change in CQ IC_50_ value for 3D7; however, CQ was more effective against K1 and NF54 in this condition, where the IC_50_ was significantly lower decreasing 3-fold each (Fig. 2). NF54 isolate showed susceptibility to CQ under both gaseous conditions; 19.3-fold more sensitive than K1, a CQ-resistant strain. In contrast to these results, under 5% O_2_ conditions, 3D7, K1, and NF54 showed atovaquone (AV) IC_50_ values between 0.11-0.37 nM, whereas under ATM the IC_50_ values had an increase (Fig. 2): approximately 3-fold for 3D7 strain and 9-fold for NF54. The apparent increase of the AV effect in K1 was not statistically relevant.

Finally, under 5% O_2_ conditions, ART did not show a statistically significant effect between the strains or the gaseous conditions, except for K1: it was possible to verify an increase in the IC_50_ value for ART in this strain, but just a 1.5-fold increase. Moreover, it was verified that the most ART-sensitive strain was NF54 followed by K1 and 3D7, under both gaseous conditions. We summarised the IC_50_ values in Table. Dose-response curves for all conditions are available in Supplementary data (Figs 1, 2). Finally, it is important to note that the introduction of low oxygen gaseous mixture returned the IC_50_ values of all antimalarial drugs to what was described by other authors.^13^ ^,^ ^20^ ^,^ ^21^

**TABLE:** Compilation of IC_50_ values. The table shows all IC_50_ values of chloroquine (CQ), atovaquone (AV), and artesunate (ART) calculated by employing the classic low oxygen mixture and atmospheric air (ATM) in 3D7 and K1 strains and NF54 isolate

	IC_50_ under 5% O_2_			IC_50_ under ATM		
	3D7	K1	NF54	3D7	K1	NF54
CQ	9.22 ± 2.32 nM	231.63 ± 43.65 nM	11.41 ± 0.70 nM	10.04 ± 0.26 nM	77.20 ± 5.64 nM	3.57 ± 0.17 nM
AV	0.37 ± 0.18 nM	0.31 ± 0.30 nM	0.11 ± 0.05 nM	1.08 ± 0.05 nM	0.69 ± 0.07 nM	0.98 ± 0.29 nM
ART	9.40 ± 2.66 nM	4.59 ± 0.53 nM	3.65 ± 1.22 nM	8.91 ± 1.96 nM	7.33 ± 0.80 nM	2.22 ± 0.52 nM

## DISCUSSION

The device of an artificial, continuous intraerythrocytic cycle *ex vivo* as described in 1976 by two groups simultaneously: Trager and Jensen^3^ and Haynes et al.^4^ Nowadays the Trager and Jensen methodology is largely the most employed in research. The method consists in maintaining statically at 37ºC candle jars and/or cell flasks containing human red blood cells suspended in a culture medium, human serum, and a gaseous mixture rich in CO_2_ and poor in O_2_,^3^ commonly 5% O_2_ and 5% CO_2_. Since 1976, several improvements to the initial protocol have been published. For example, Moloney et al.^10^ developed culture conditions to enable the asexual erythrocytic stage of *P. falciparum* to be grown in deep culture (several centimetres depth) in reusable bottles gassed with 5% oxygen, 5% carbon dioxide, and 90% nitrogen. Thus, the authors published a protocol that makes it easier and less expensive to cultivate large volumes of parasites in a single vessel (up to eight litres).^10^ Here, we showed a continuous culture of *P. falciparum* in a shaker with reusable-glass bottles but employing ATM (approximately 78.09% N_2_, 20.95% O_2_, 0.93% CO_2,_ and 0.04% other residual gases). With this methodology, it is also possible to easily cultivate large volumes of highly parasited cultures (20 to 30% parasitaemia) and to obtain high parasitic yields, 1x10^10^ parasites in a few 1-2 L bottles (data not shown).

The use of ATM did not affect substantially the proliferation of parasites in comparison to the Trager and Jensen low-oxygen gaseous mixtures.^3^ Conceptually, the use of low-oxygen mixtures (~ 5% O_2_) was defined to be normoxic, regarding the natural gaseous composition of tissues.^3^ Thus, a 5% O_2_ environment is applied to culture vessels and/or incubators. However, the pericellular environment of the red blood cells may not present the same gaseous conditions. Before oxygen reaches the cells, it must be exchanged at the liquid-gas surface and dissolved in the culture medium in a process that is dependent on several factors such as pH, cell density, pressure, and medium volume and composition.^22^ Notably, oxygen travels a few millimetres to reach cells in culture, whereas it travels approximately 10-30 μm in animal tissues.^23^ Once the cells are consuming oxygen, its diffusion must exceed consumption to avoid hypoxia. Considering all this, Branco et al., previously argued that it is difficult to reproduce physiologic-like conditions *in vitro*.^11^ The authors mentioned large differences between the O_2_ saturations across human tissues, where some tissues can experience 10-13% O_2_ saturation.

To exactly determine O_2_ levels in culture, we did oxygraphy assays. Considering that arterial oxygen concentration is approximately 130 µM,^22^ our results indicate that the parasite statically cultured *in vitro* is exposed to slightly hyperoxia by employing both ATM and classic 5% O_2_ conditions. It should be noted that we assessed the free oxygen content in homogenised uninfected erythrocytes; thus, the actual oxygen concentration reaching erythrocytes at the bottom of the flask is probably lower, quickly consumed by parasites, or bound to haemoglobin. In fact, in cytotrophoblast cell cultures *in vitro*, a four-fold decrease in O_2_ concentration at the bottom of the flask compared with the gas-liquid interphase has been previously reported.^24^ Similar to what Branco et al., mentioned,^11^ here we propose that the use of oxygen-rich mixtures may create a pericellular O_2_ concentration more similar to the natural environment of cells.

Irrespectively of the O_2_ levels available for *Plasmodium* cultured *in vitro*, it should be noted that ATM only decreased parasite viability at very low parasitaemia levels (< 0.5%) but did not affect proliferation rates or the proportion of the parasitic stages above that limit. These low parasitaemia conditions are rarely employed in experiments but may limit the use of ATM in drug screening assays by (^3^H) hypoxanthine incorporation or DNA staining.^12^ ^,^ ^19^ ^,^ ^25^ Nevertheless, cultures of *P. falciparum* 3D7 strain have been maintained under ATM in our group for years, which showed the suitability of the technique for use over extended periods.

An important requirement for *in vitro* culture is the ability to mimic physiologic conditions that can allow meaningful extrapolations of the biological reality. Nevertheless, there are factors, such as temperature changes and immune responses, that are not considered regularly, although in some instances they may induce relevant differences between *Plasmodium* cultures and the human infection.^11^ Probably, the most controversial factor reported in the literature about *Plasmodium* cultures is the *in vitro* gaseous composition employed.^11^

**Fig. 3:**
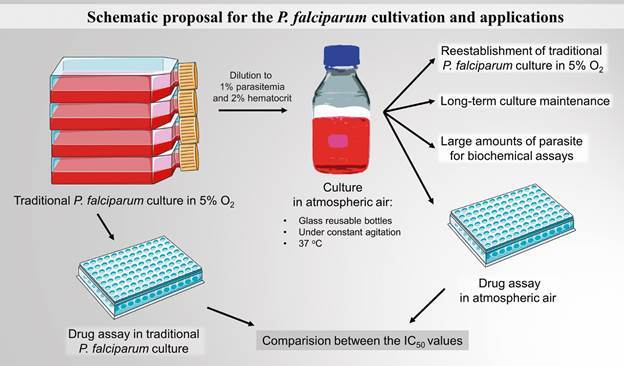
schematic proposal for *Plasmodium falciparum* cultivation. The figure shows a schematic proposal for the *P. falciparum* cultivation as it is better described in the text using images from Servier Medical Art (licensed under a Creative Commons Attribution 3.0 Unported Licence).

Different gaseous mixtures and ATM were employed across the studies and marginal effects on the parasitic viability were observed.^12^ ^,^ ^13^ ^,^ ^26^ ^-^ ^32^ The major effect reported on growth characteristics derived from modifying gaseous conditions is a little effect with an increase in the length of schizogony in parasites exposed to ATM.^13^ However, in *in vitro* pharmacological studies, parasites cultured at high oxygen tensions also showed different susceptibility to some antimalarials.^13^ ^,^ ^14^ This made some authors suggest the necessity of a standardised *in vitro* assay protocol to survey malaria drug resistance.^13^ In particular, Briolant et al., showed that CQ IC_50_ values obtained at 10% O_2_ were significantly higher than those found in several isolates maintained at ATM.^13^ The authors showed that 30% of the isolates that were *in vitro* resistant to CQ (IC_50_ > 100 nM) at 10% O_2_, become sensitive (IC_50_ < 100 nM) at ATM, our results were consistent with this. Also, Duffy & Avery 2017 showed that parasites cultured for several months in media supplemented with a serum substitute or within hyperoxic conditions demonstrate variable responses to artemisinin and lumefantrine.^30^ Mirovsky in 1989^12^ observed a significantly lower parasitaemia within the first five days of cultivation under ATM and failed at cultivating in Petri dishes without performing the candle jar technique. However, here we further show that cultivation under ATM is suitable in cell culture flasks and glass bottles at high parasitaemia levels. From a biochemical point of view, Torrentino-Madamet et al.,^33^ detected transcriptional changes in parasites maintained in hyperoxic conditions. Specifically, the authors detected an up-expression of genes involved in antioxidant systems and a down-expression of genes involved in the digestive vacuole metabolism and the glycolysis in favor of mitochondrial respiration. Furthermore, the authors also demonstrated increased levels of heat shock proteins and decreased levels of glycolytic enzymes in parasites maintained in hyperoxic conditions. Torrentino-Madamet et al., explain their results as a natural and efficient metabolic adaptability to varying oxygen pressures in different hosts or localisations within the human body.^33^ According to this, we show that variable responses to antimalarial drugs CQ and AV due to hyperoxic conditions are reversed in the first cycle if parasites are cultured again in low oxygen environments. Further, our group has previously employed non-classical gaseous mixtures (0% or 20% O_2_) for biochemical purposes.^26^ ^,^ ^27^ ^,^ ^28^ In both cases, we observed parasite viability for at least two-three weeks under different gaseous mixtures.^26^ ^,^ ^27^ ^,^ ^28^ ^,^ ^29^ Therefore, ATM conditions and shaken flasks could be used as a standard method condition for culture manutention, since they do not differ greatly from classical 5% O_2_ gas mixtures or the probable natural conditions in terms of parasite proliferation and do not impose non-reversible changes to *P. falciparum* physiology. It remains poorly studied why some antimalarials become more or less effective in the function of the O_2_ content. For example, here we showed that changes in the oxygen tension can modulate AV and CQ effects, but not ART, in different directions and proportions across the strains. From a biochemical perspective, differences in the antimalarial effects under different gaseous conditions can be interesting tools to better understand the parasitic metabolism and mechanisms of action of different drugs. Finally, to summarise this article, we show a schematic proposal for *P. falciparum* cultivation and its applications (Fig. 3).
