# Supplementary material for: Suitability of methods for Plasmodium falciparum cultivation in atmospheric air
Source: Mem Inst Oswaldo Cruz. 2022 Jul 18;117:e210331. doi: 10.1590/0074-02760210331 (PMC9296140; doi:10.1590/0074-02760210331)
Supplement: Supplementary file 1 [file 1678-8060-mioc-117-e210331-s.pdf]

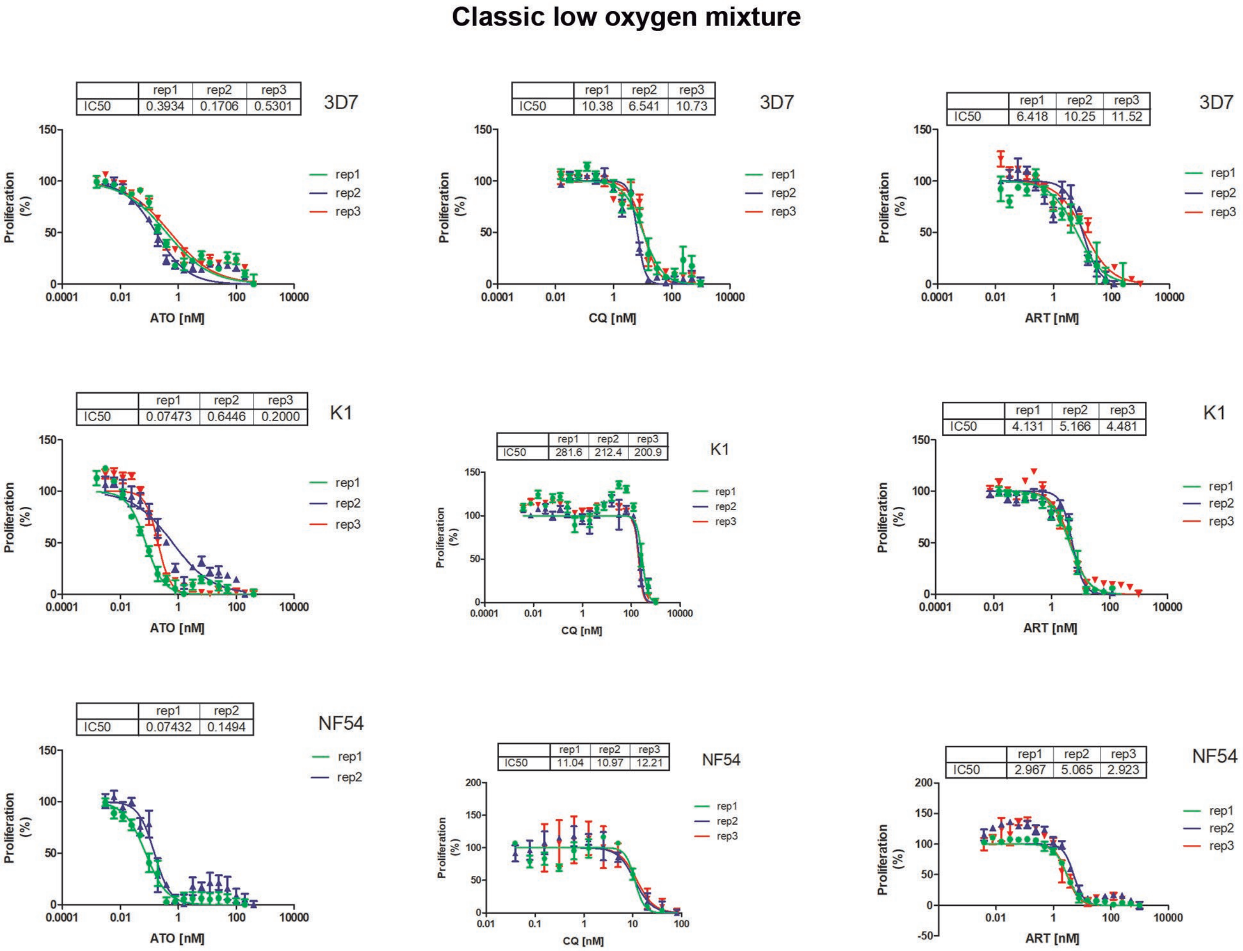

Fig. 1: sigmoidal dose-response effect of antimalarials under classic low oxygen mixture. The sigmoidal dose-response effect of atovaquone (AV), chloroquine (CQ), and artesunate (ART) in *Plasmodium falciparum* 3D7, K1, and NF54 isolates utilising a classic gaseous mixture established by Trager and Jensen. The parasites were cultivated for 48 h at 37ºC in 96 well plates.

CC BY

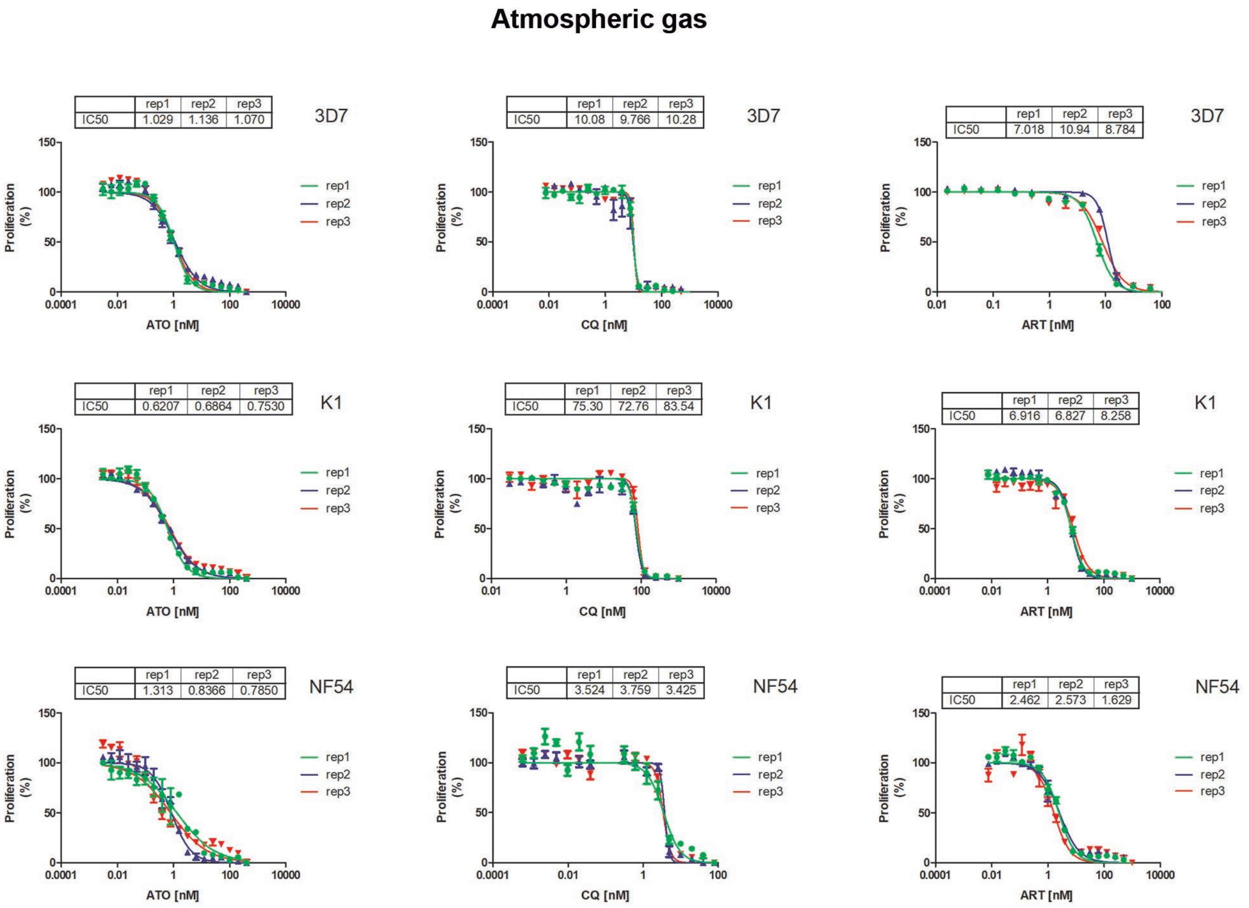

Fig. 2: sigmoidal dose-response effect of antimalarial under atmospheric air (ATM). The sigmoidal dose-response effect of atovaquone (AV), chloroquine (CQ), and artesunate (ART) in *Plasmodium falciparum* 3D7, K1, and NF54 isolates without adding a gaseous mixture. The parasites were cultivated for 48 h at 37ºC in 96 well plates.
